# Low-level Gestational Lead Exposure Alters Dendritic Spine Plasticity in the Hippocampus and Reduces Learning and Memory in Rats

**DOI:** 10.1038/s41598-018-21521-8

**Published:** 2018-02-23

**Authors:** Zai-Hua Zhao, Gang Zheng, Tao Wang, Ke-jun Du, Xiao Han, Wen-Jing Luo, Xue-Feng Shen, Jing-Yuan Chen

**Affiliations:** Department of Occupational and Environmental Health and the Ministry of Education Key Lab of Hazard Assessment and Control in Special Operational Environment, School of Public Health, Fourth Military Medical University, No 169 of West Changle Road, Xi’an, Shaanxi 710032 China

## Abstract

Lead (Pb) is known to impair children’s cognitive function. It has been previously shown that developmental Pb exposure alters dendritic spine formation in hippocampal pyramidal neurons. However, the underlying mechanism has not yet been defined. In this study, a low-level gestational Pb exposure (GLE) rat model was employed to investigate the impact of Pb on the spine density of the hippocampal pyramidal neurons and its regulatory mechanism. Pb exposure resulted in impaired performance of the rats in the Morris water maze tasks, and in decreased EPSC amplitudes in hippocampal CA3-CA1 regions. With a 3D reconstruction by the Imaris software, the results from Golgi staining showed that the spine density in the CA1 region was reduced in the Pb-exposed rats in a dose-dependent manner. Decreased spine density was also observed in cultured hippocampal neurons following the Pb treatment. Furthermore, the expression level of NLGN1, a postsynaptic protein that mediates synaptogenesis, was significantly decreased following the Pb exposure both *in vivo* and *in vitro*. Up-regulation of NLGN1 in cultured primary neurons partially attenuated the impact of Pb on the spine density. Taken together, our resultssuggest that Pb exposure alters spine plasticity in the developing hippocampus by down-regulating NLGN1 protein levels.

## Introduction

Lead (Pb) is a widespread environmental heavy metal toxicant that exerts irreversible effects on children’s cognitive function, impairing learning and memory^1^, decreasing intelligence quotient^2^, and increasing aggression and crime rates^3^. Lead can enter the human body via skin, respiratory system and/or gastrointestinal tract^4^, and the regulatory blood-lead-level (BLL) thresholds for childhood Pb poisoning have gradually decreased, from 30–60 μg/dl in 1975 to 5 μg/dl in 2012^5^. However, recent studies have shown that BLL as low as 1–5 μg/dl poses a risk for neurocognitive effects in the fetus and newborn^6^. Furthermore, some studies have suggested that there is no safe threshold for Pb exposed children^7–9^. The developing brain, especially hippocampus, is particularly vulnerable to Pb. Due to the central role of the hippocampus in spatial learning and memory, numerous studies have focused on Pb’s effects on hippocampal-associated spatial learning and memory processes. It has been observed that developmental Pb exposure in rats decreases the frequency and amplitude of hippocampal long-term potentiation (LTP)^1,10^, which represents a physiological model of learning and memory. Additionally, morphological analyses have shown a reduction in dendritic spine density in hippocampal CA1 and DG neurons upon developmental Pb exposure^11,12^.

The brain is exquisitely sensitive to Pb during early developmental stages, especially throughout the gestational period when key developmental processes, such as neuron proliferation, differentiation, migration and synaptogenesis, occur. Current rodent models of Pb exposure have mostly focused on perinatal exposures^13,14^, early postnatal exposures^15^ and late postnatal exposures^16^. To date, it has been shown that Pb exposure impairs hippocampal-dependent spatial learning and memory by altering *N*-methyl-D-aspartate (NMDA) receptor-dependent brain derived neurotrophic factors (BDNF)^17^, by impairing hippocampal LTP, possibly via microglia-neuron cross-talk^18^, and by introducing epigenetic changes through manipulating the expression of DNA methyltransferases and methyl cytosine-binding proteins^19^.

Dendritic spines, the main structures of pyramidal neurons, are the sites harboring the post-synaptic compartment of excitatory synapses^20,21^. Changes in dendritic spine density and structure are crucial for post-synaptic plasticity and contribute to the morphological bases of learning and memory function^22,23^. The induction of spine growth often coincides with synapse formation^24^. Synaptic formation, structure, maturation and maintenance are sustained by a diverse network of bidirectional cellular adhesion molecules that span the synaptic cleft, aligning the presynaptic active zone and postsynaptic density^25^. In humans, alterations that perturb these cellular organizations are implicated in cognitive disorders, highlighting their critical roles at the synapse. Arguably, one of the best-characterized synaptic cell adhesion molecules is the postsynaptic neuroligins (NLGNs)^26,27^. Neuroligins are postsynaptic proteins that contain a single transmembrane domain, a large extracellular acetylcholinesterase-like domain and a short cytoplasmic tail (c-tail) that includes many protein-protein interaction domains^21,28^. Rodents express four neuroligins (NLGN1 to NLGN4). Neuroligin 1 (NLGN1), a postsynaptic adhesion protein, is located predominantly in excitatory synapses and is involved in diverse forms of excitatory synaptic plasticity across species^29,30^. Overexpression of NLGN1 increases evoked excitatory postsynaptic currents (EPSCs) and the number of excitatory synapses, whereas silencing NLGN1 reduces the density of excitatory synapses^31,32^. NLGN proteins are expressed in both the developing and mature brain. NLGN proteins, especially NLGN1, are required for synaptogenesis, dendritic spine maturation and stability. Taken together, these observations have led us to postulate that developmental Pb exposure disrupts NLGN1, leading to altered dendritic spine formation in hippocampal neurons, which, in turn, results in learning and memory impairment. Given that gestational brain development in humans is analogous to the prenatal through PND10 period in rats^33–35^, we established a low-level human-equivalent gestational Pb exposure (GLE) rat model to explore this issue.

## Results

### Low-level gestational Pb exposure impaired spatial learning and memory

**Figure 1:**
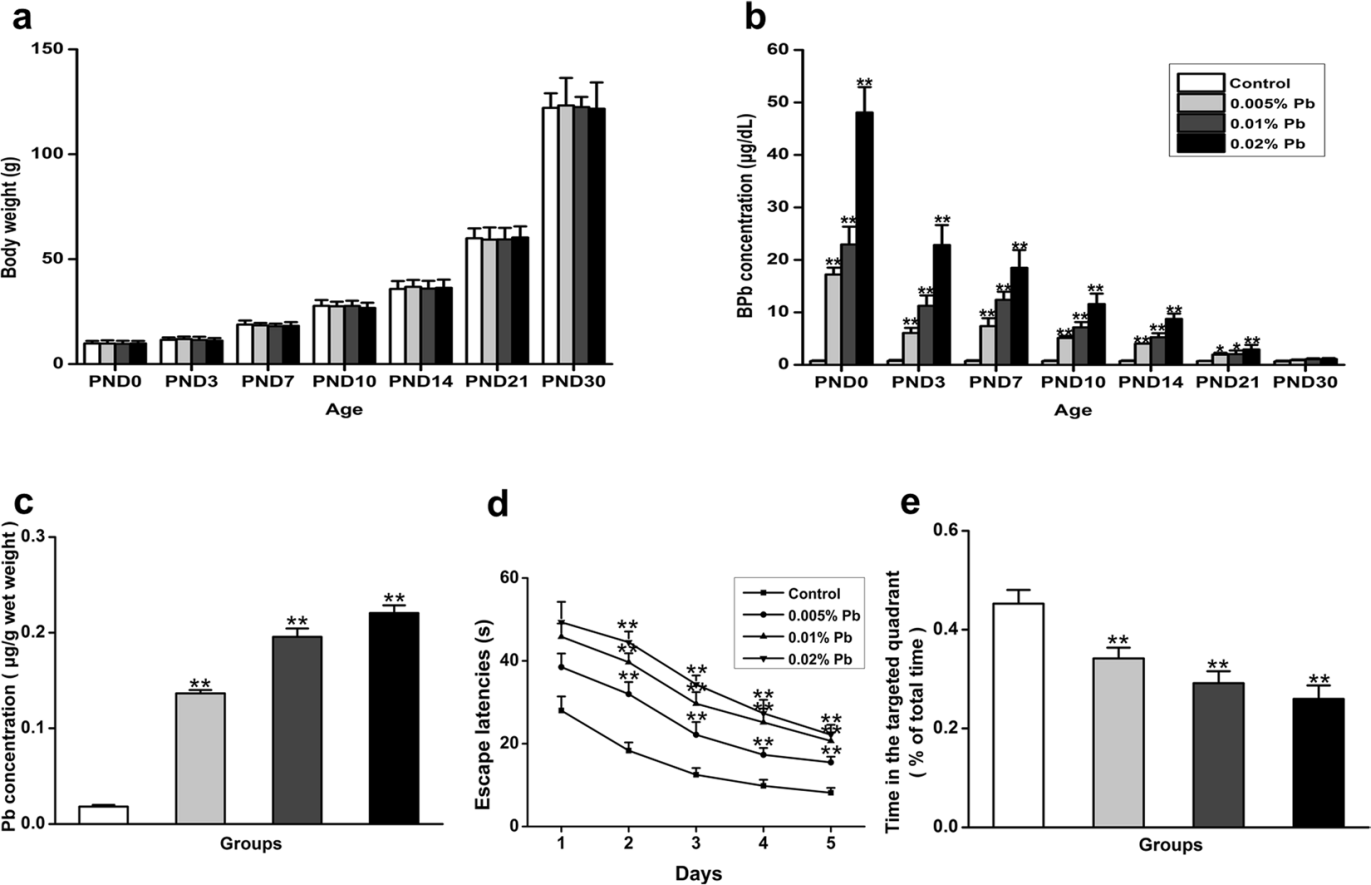
Pb accumulation in the hippocampus and its effect on learning and memory on PND30 rats. Morris water maze test was performed to detect learning and memory ability in four groups of animals: Control, 0.005% Pb, 0.01% Pb and 0.02% Pb. (**a**) Body weights (N = 8 per group) and (**b**) blood-lead-levels (BLLs) (N = 8 per group) were measured at postnatal day (PND) 0, 3, 7, 10, 14, 21 and 30. (**c**) Hippocampal Pb levels (N = 8 per group) and (**d**,**e**) the water maze test (N = 8 per group) were only measured at PND 30. Test results showed that with increased Pb exposure, the escape latencies were positively correlated, and time spent in the targeted quadrant at the test day were negative correlated. Data are expressed as mean ± SD, *p < 0.05, **p < 0.01.

Female SD rats from each groups (respectively, n = 8) were exposed to water containing 0 (Control), 0.005%, 0.01% and 0.02% Pb acetate for two weeks before mating and throughout gestation. Pb exposure ceased 10 days after the progeny were born. Figure 1a shows the body weights of pups at PNDs 0, 3, 7, 10, 14, 21, and 30. There were no significant effects of GLE on body weight at any age (Fig. 1a). Blood and hippocampal Pb levels were determined by a graphite furnace atomic spectrophotometry. BLLs of the pups were significantly increased in the Pb-exposed groups at PNDs 0, 3, 7, 10, 14 and 21, except for PND 30 (Fig. 1b). Hippocampal Pb levels of the pups were significantly increased in the Pb-exposed group at PND 30 (Fig. 1c). Spatial learning and memory of the pups were assessed by the water maze task at PND 30 (Fig. 1d,e), which measures the time spent in finding an invisible platform. Searching time for the platform was longer in the Pb-exposed rats when compared with the controls throughout the five training days (Fig. 1d). On the day for the probe trial, the platform was removed. The percentage of time spent in exploring the platform in the southwest (SW) quadrant, where the platform was originally located, was significantly lower in any Pb-exposed groups than that in the control group (Fig. 1e), demonstrating impaired spatial memory in Pb-exposed rats.

### Low-level gestational Pb exposure impaired hippocampal CA3-CA1 Long-Term Potentiation

**Figure 2:**
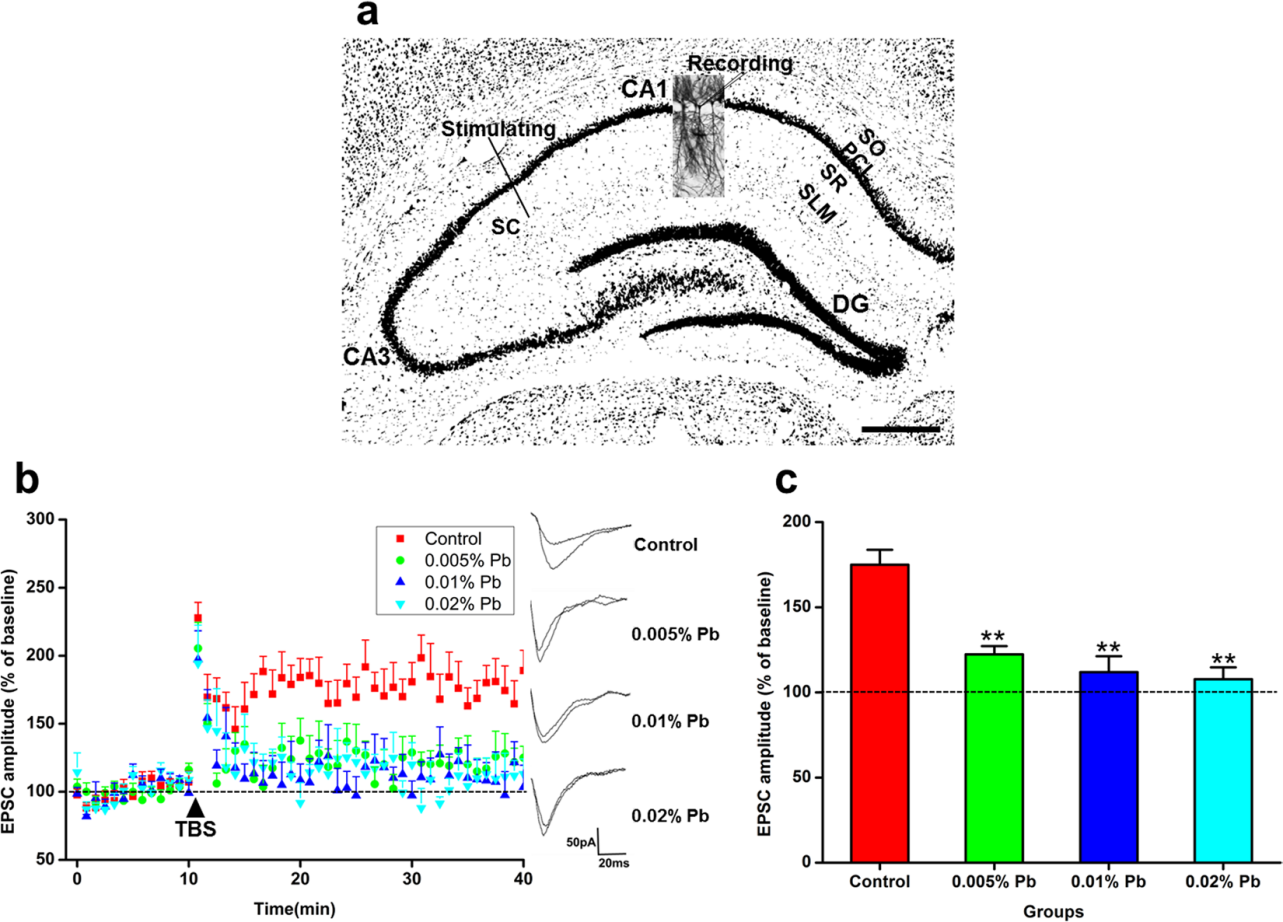
Pb accumulated in the hippocampus and impaired CA3-CA1 LTP of Pb-exposed PND30 rats. (**a**) Simulations were applied to the SC regions of hippocampus and the signal was collected at the CA1 region. stratum oriens, SO; pyramidal cell layer, PCL; stratum radiatum, SR; stratum lacunosum-moleculare, SL-M; dentate gyrus, DG; schaffer collaterals, SC; scale bar: 500 *μ*m. (**b**) LTP in hippocampal regions CA3-CA1. Arrowhead indicates time of tetanic stimulation. The EPSC amplitude showed a negative correlation between Pb exposure and LTP. N = 16 slices of 8 rats for all groups. (**c**) EPSCs were measured as percentage of baselines, respectively. Statistical analysis of LTP at PND 30 showed that GLE rodents exposed to 0.02% Pb showed the lowest EPSC amplitude. Data are expressed as mean ± SD, **p < 0.01.

Rats were decapitated between PND 28 and 35, and the hippocampal slices of each groups were subjected to LTP induction. After obtaining a 10-min baseline response of EPSC, LTP was induced by high frequency tetanic stimulation (Fig. 2a). In the Pb-exposed groups, the magnitudes of EPSCs were lower than those in the control group (Fig. 2b). The increase in the EPSCs amplitudes (as a percentage of the baseline) decreased in any Pb-exposed groups compared with the control (Fig. 2c). These results suggested that hippocampal CA3-CA1 LTP inductions were significantly impaired in the Pb-exposed rats.

### Pb exposure did not affect dendritic complexity of the pyramidal neuron in PND30 rats

**Figure 3:**
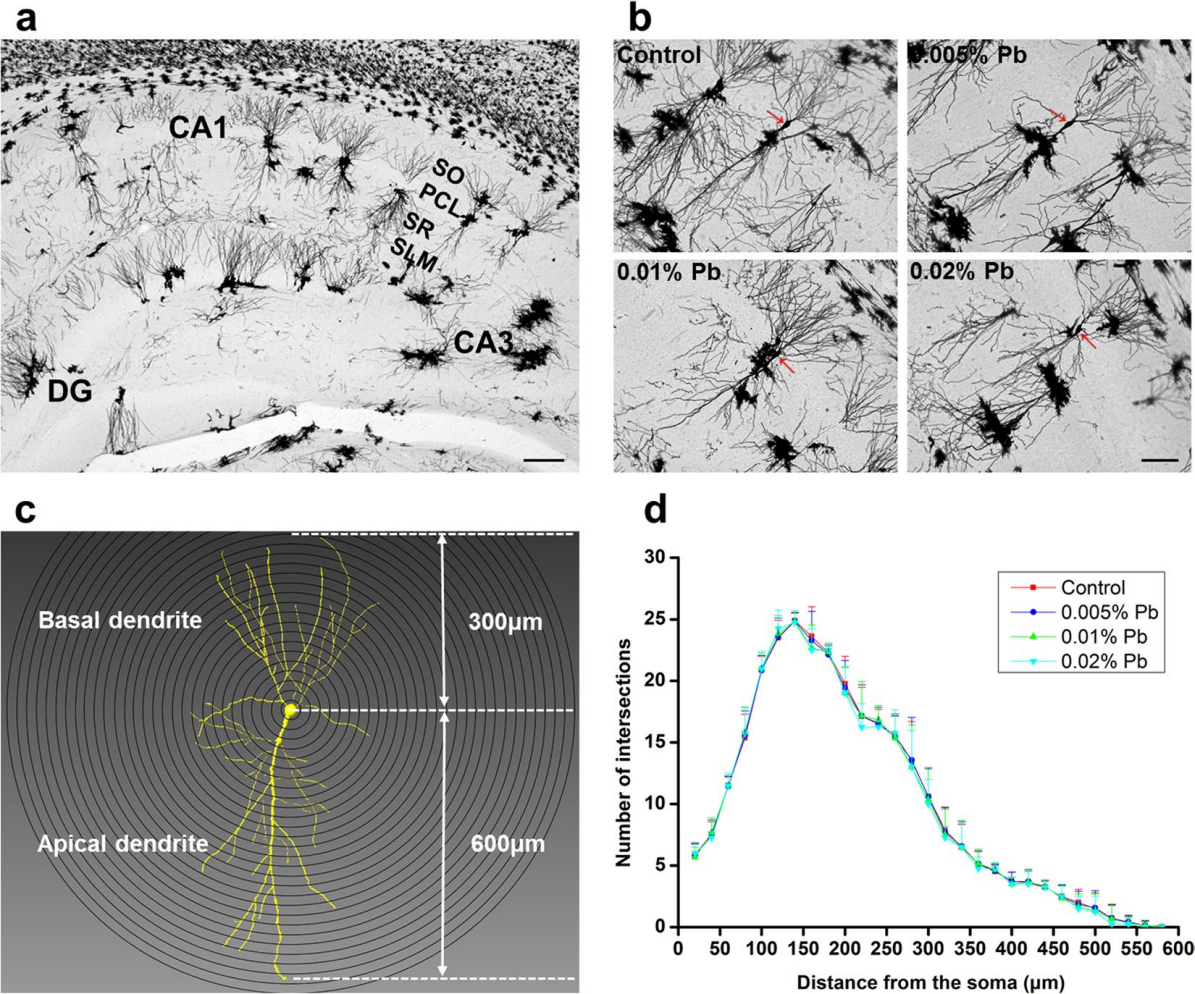
Dendritic morphology quantified with 3D reconstructions of Golgi impregnated neurons and software-assisted tracing of entire dendritic arbors of stained neurons. (**a**) Golgi Staining of rat hippocampus. stratum oriens, SO; pyramidal cell layer, PCL; stratum radiatum, SR; stratum lacunosum-moleculare, SL-M; dentate gyrus, DG; scale bars = 200 μm. (**b**) Golgi staining of CA1 pyramidal neurons, scale bars = 100 μm. (**c**) Imaris reconstruction of CA1 pyramidal neurons (16 neurons/8 rats for all groups). (**d**) Sholl analysis was applied to measure the dendritic complicity of reconstructed pyramidal neurons, which showed no differences in the numbers of intersections for all groups of GLE Pb-treated rodents. Data are expressed as mean ± SD.

Pyramidal neurons in the hippocampal CA1 region were shown by Golgi Staining (Fig. 3a,b). Dendritic morphology was qualified by 3D reconstructions of Golgi impregnations and software-assisted tracing of entire dendritic arbors of stained neurons (Fig. 3c). Sholl analysis revealed that the intersection number of the dendritic arborizations was not significantly changed between the control and Pb-exposed groups (Fig. 3d). These results indicated that dendritic development was unaffected by low-level gestational Pb exposure.

### Pb exposure impaired dendritic spine formation and maturation of pyramidal neuron in PND30 rats

**Figure 4:**
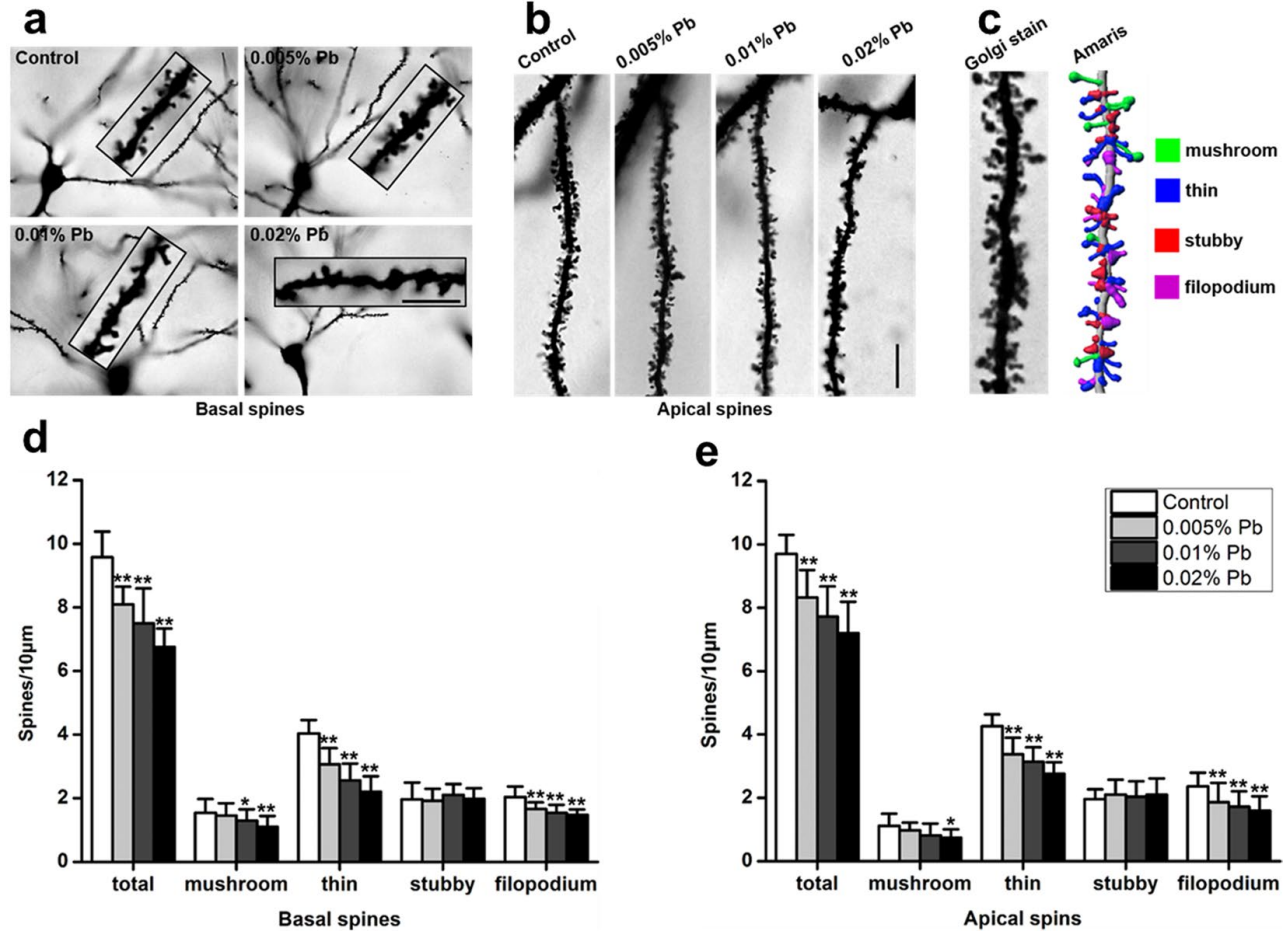
Pb exposure reduced the density of dendritic spines of pyramidal neurons in PND30 rats. (**a**) Golgi staining of basal spines, scale bars = 5 μm. (**b**) Golgi staining of apical spines, scale bars = 10 μm. (**c**) Imaris reconstruction of spines. (**d**) Statistic analysis of the effect of Pb exposure on basal spines (16 neurons/8 rats for all groups). (**e**) Statistic analysis of the effect of Pb exposure on apical spines (16 neurons/8 rats for all groups). Data are expressed as mean ± SD of spines per 10 μm for all groups of rats, *p < 0.05, **p < 0.01.

We assessed whether the functional alterations were accompanied with the density and morphological changes in the dendritic spines of CA1 pyramidal neurons. First, we reconstructed and analyzed the dendritic spines of CA1 pyramidal neurons with the Imaris software (Fig. 4c). The average spine densities in the Pb-treated groups significantly decreased as the dose of Pb exposure increased, in both basal and apical dendritic spines, compared with those in the control group (Fig. 4d,e). In the basal dendritic spines, densities of thin and filopodium spines were significantly reduced in all the Pb-exposed groups. However, the density of mushroom spines decreased only in the 0.01% and 0.02% Pb-exposed groups compared with the control group (Fig. 4d). In apical dendritic spines, densities of thin and filopodium spines were also reduced in all the Pb-exposed groups, while the density of mushroom spines decreased only in the 0.02% Pb-exposed group compared with the control group (Fig. 4e). These results established that Pb exposure significantly impaired spine formation in the developing hippocampus.

### Low-level gestational Pb exposure reduced NLGN1 mRNA and protein expression in PND30 rats

**Figure 5:**
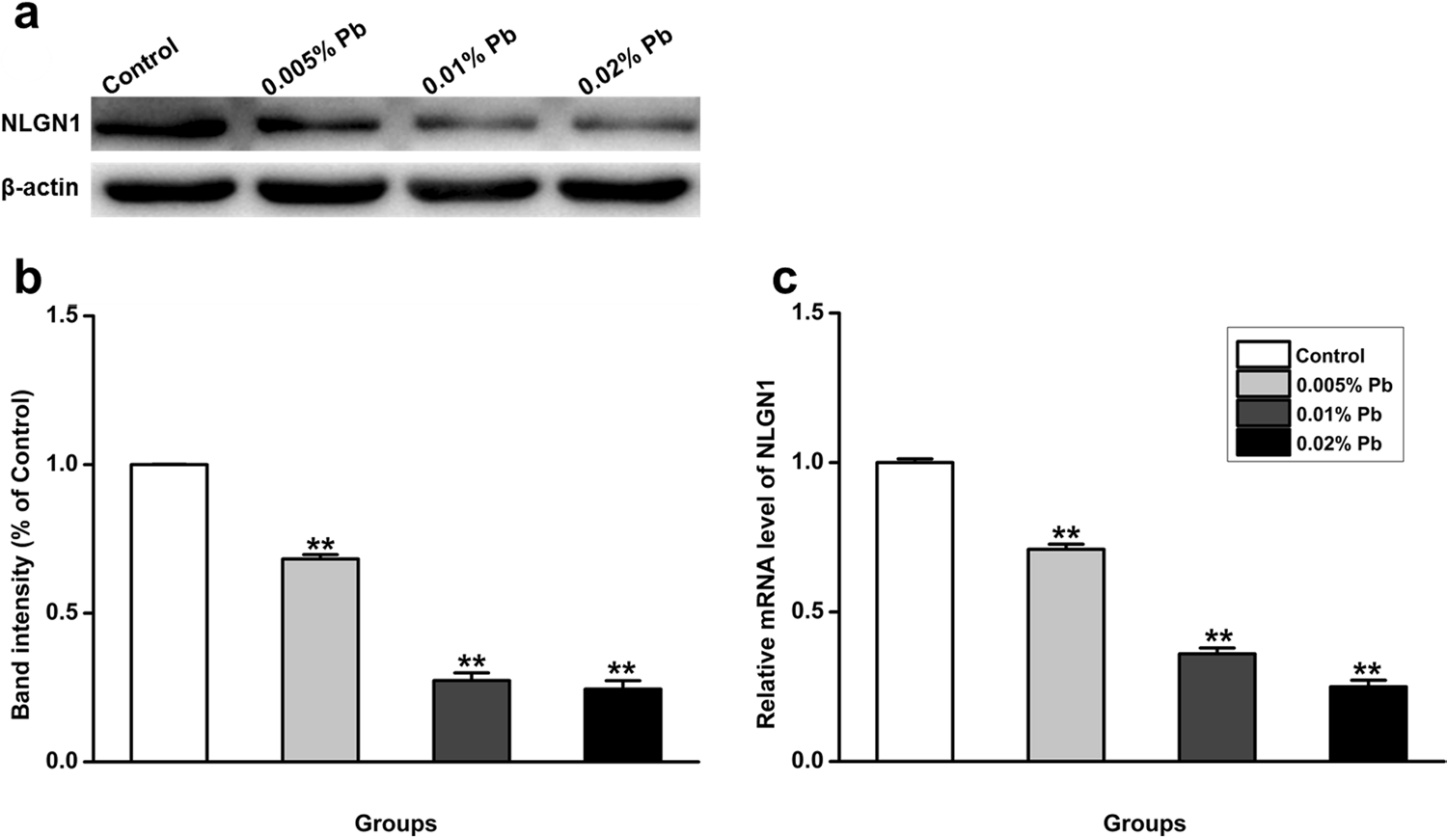
Effect of Pb exposure on NLGN1 transcript and protein expression in PND30 rats. (**a**) Protein expression of NLGN1 after Pb exposure was detected by western blotting.β-actin was detected as loading control. N = 8 per group. (**b**) Statistical analysis of the band intensity, which was measured as percentage of control. The protein level of NLGN1 is Pb dose-depended, negatively correlated with the Pb exposure levels. (**c**) NLGN1 mRNA expression was detected in the control group and Pb-exposed groups by qRT-PCR. The mRNA level of NLGN1 is Pb dose-depended, negatively correlated with the Pb exposure levels. N = 8 per group. Data are representative of at least three independently performed experiments and expressed as mean ± SD, **p < 0.01.

Considering the important roles of NLGN1 in the formation and regulation of the spines in the hippocampus, we then examined whether the Pb-induced decrease in spine density in the CA1 region was accompanied by a reduction in NLGN1 expression. NLGN1 protein levels were significantly reduced in rats after low-level gestational Pb exposure (Fig. 5a,b). Furthermore, Real-time fluorescence quantitative PCR reveals that the NLGN1 mRNA level in Pb-exposed rats were down-regulated in all groups compared with the control group (Fig. 5c).

### NLGN1 overexpression rescued the Pb-induced reduction in spine density

**Figure 6:**
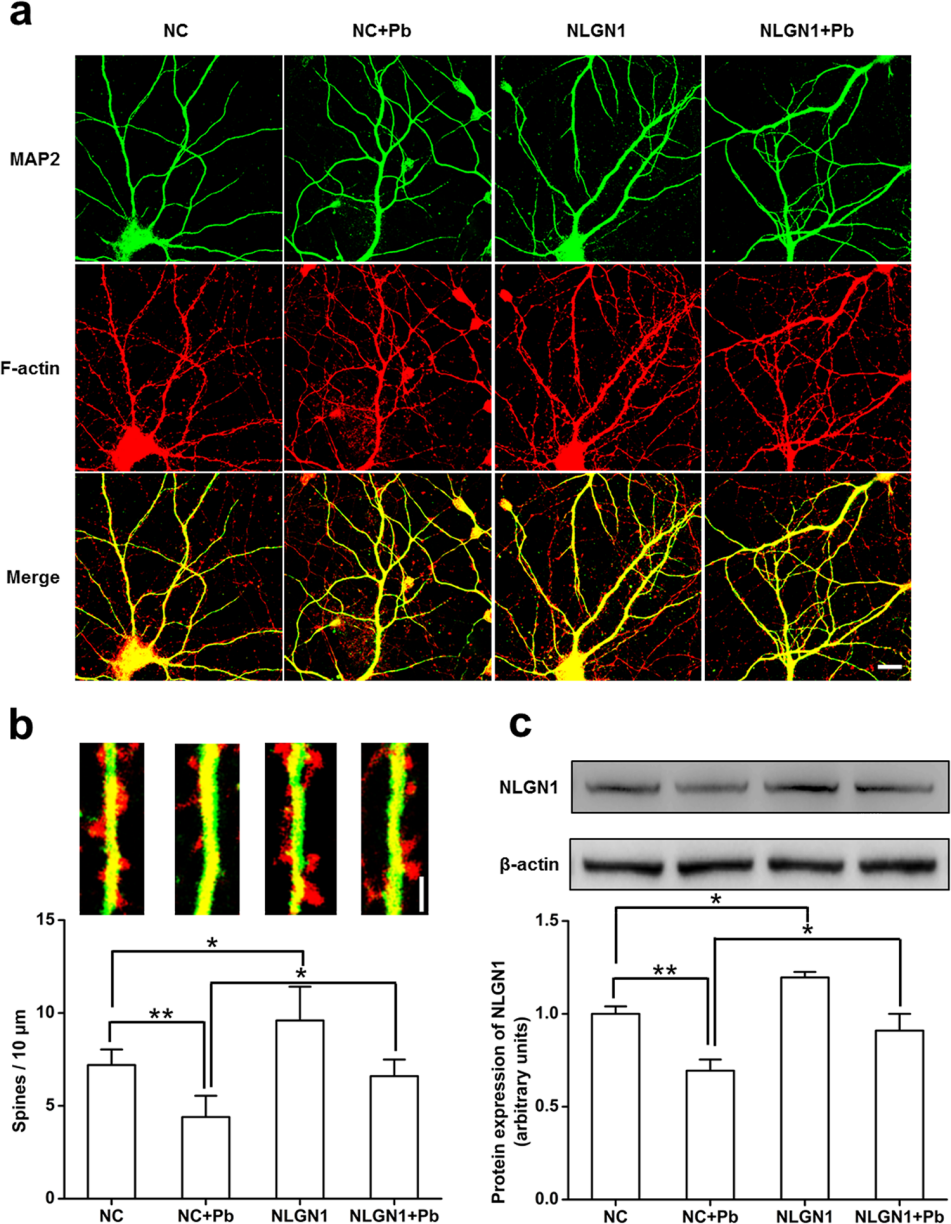
Effect of NLGN1 overexpression on dendritic spine density in hippocampal pyramidal neurons upon Pb treatment *in vitro*. (**a**) primary cultured neurons stained with MAP2 and F-actin, scale bar = 20 μm. N = 16 neurons per group. (**b**) Top: from left to right, are NC, NC + Pb, NLGN1 and NLGN1 + Pb groups, respectively, scale bar = 5 μm; bottom: statistical analysis of spine densities after Pb exposure. Data are representative of at least three independently performed experiments and expressed as mean ± SD of spines per 10 μm for all groups, *p < 0.05, **p < 0.01. (**c**) Up regulation of NLGN1 was detected via Western Blot. Overexpression of NLGN1 led to increased expression of NLGN1 protein in GLE rats. Data are representative of at least three independently performed experiments and expressed as mean ± SD, *p < 0.05, **p < 0.01.

Since NLGN1 is required for excitatory synapse formation, we explored whether Pb affects spine formation by down-regulating NLGN1 expression. NLGN1 was over-expressed in primary cultured hippocampal neurons to examine its ability to rescue the Pb-induced decline in dendritic spine density (Fig. 6c). Up-regulation of NLGN1 level was associated with a significant increase in spine density in the Pb-treated group, increasing to 86.53% of control non-exposed cells (Fig. 6a,b). These data suggested that NLGN1 is critical in mediating the early structural effects of Pb on synaptogenesis.

## Discussion

This study investigated the impact of low-level gestational Pb exposure on learning and memory and the role of NLGN1 in this process. We report on several novel findings. First, hippocampal Pb levels were significantly increased and hippocampal LTPs were significantly impaired despite BLLs in the Pb-exposed groups at PND30 were indistinguishable from controls. Second, offspring of GLE dams exhibited alterations in dendritic spine density and morphology in hippocampal CA1 pyramidal neurons. Finally, overexpression of NLGN1 rescued the Pb-induced reduction of spine density in hippocampal neurons.

The rats were exposed to Pb for two weeks prior to mating in order to keep a steady Pb body burden in the females; the Pb exposure was ceased 10 days after the progeny were born, because the gestational period plus the first 10 days of neonatal life in the rat are equivalent to the human gestational period^33^. After GLE, the BLL returned to normal levels in all PND30 animals, yet the hippocampal Pb level remained significantly higher and spatial learning and memory abilities were significantly impaired in the Pb-exposed rats. Our previous work demonstrated that Connexin43 (Cx43) hemichannels were involved in Pb transport from extracellular to intracellular compartments^36^. Futhermore, Cx43 expression was increased in the hippocampus, one of the major targets of Pb-induced neurotoxicity, in Pb-exposed rats^37^. These studies suggest that Pb might accumulate in the hippocampus via the Cx43 hemichannel. Our findings are consistent with clinical observations, which corroborate that chelation therapy lowering of BLLs, but the therapy fails to reverse the cognitive deficits in children even after BLLs return to normal levels^38^.

Our studies showed that Pb-exposed rats had deficits in the Morris water maze tasks, consistent with impaired learning and memory. Synaptic plasticity in the hippocampus is a crucial neurobiological basis for learning and memory. Previous studies on Pb neurotoxicity mainly focused on functional plasticity of the synapse^17,18^; however, the changes in functional plasticity cannot completely explain the mechanisms underlying Pb neurotoxicity. As structural plasticity is the basis of functional plasticity of the synapse, structural changes of synapses may also be an important cause of Pb-induced learning and memory dysfunction. Plasticity of dendritic spines, including changes in the number and shape of dendritic spines, plays a significant role in the excitatory synaptic transmission and is closely associated with learning and memory. Although the Pb exposure did not resulted in a significantly change in the dendritic complexity, as previously reported^1^, the average spine density in the Pb-treated groups was significantly decreased in a dose-dependent manner in both basal and apical dendritic spines compared with the control group. A notable strength of our work is that we separately analyzed basal and apical spines, unlike previous work^1,11^. By categorizing the spines into four types (mushroom, thin, stubby and filopodium) with the Imaris software^39^, we noted that thin and filopodium densities were significantly reduced in all Pb-exposed groups. The mushroom densities were significantly reduced in 0.01% and 0.02% Pb-exposed groups for basal dendritic spines and significantly reduced in the 0.02% Pb-exposed groups for apical dendritic spines.

Common hypothesis posit that filopodium spines are the precursors of other dendritic spines^40,41^. Accordingly, a reduction in the density of the filopodium spines suggests the impairment in spine formation. Thin spines, with a long neck and a clearly visible small head, are dynamic and believed to be closely associated with learning abilities^42^. Mushroom spines are steadier and mature spines forming synapses; in other studies, a decrease in the number of mushroom spines was noted to be closely associated with functional loss of long- and short-term memories^43^. Thus, the decrease in thin and mushroom spine density observed herein may contribute to the impairment of learning and memory following gestational Pb exposure.

Another goal of our study was to explore the molecular mechanism underlying GLE induced reduction of dendritic spines. Previous research has demonstrated that developmental lead exposure altered synaptogenesis through inhibiting canonical Wnt pathway *in vivo* and *in vitro*^11^. A recent study showed that the activity-regulated cytoskeletal-associated protein (Arc/Arg3.1), which was widely implicated in synaptic plasticity and hippocampal dependent memory formation, might have a critical role in the disruption of neuronal morphology and synaptic plasticity in lead-exposed rats^12^. In our study, we focused on neuroligin 1 (NLGN1), a postsynaptic adhesion protein, which is located predominantly on the excitatory synapses and participates in diverse forms of excitatory synaptic plasticity across species^29,30^. Previous studies have found that overexpressing NLGN1 promoted synapse formation, whereas silencing NLGN1 reduced synapse number^31,44–46^. In other studies, NLGN1 KO rats showed a reduction in LTP plasticity and decreased learning and memory ability combined with cognitive dysfunction^29,32^. These findings suggested that NLGN1 may play a vital role in the synapse formation and LTP induction. In the present study, GLE caused a decrease in hippocampal NLGN1 mRNA and protein levels both *in vivo* and *in vitro*, and overexpression of NLGN1 partially rescued the Pb-induced decrease in dendritic spine density. Thus, GLE-induced reduction of NLGN1 expression is likely to be a major cause of the decrease in spine density in pyramidal neurons, and the ensuing altered LTP formation. Further *in vivo* studies are necessary to investigate the effects of NLGN1 up-regulation on attenuating the Pb-induced impairment of learning and memory.

The regulatory mechanism by which Pb down-regulated the expression of NLGN1 remains largely unknown. Former studies have demonstrated that DNA methylation may interrupt the DNA transcription, resulting in changes in the expression of mRNA and protein levels. *In vitro* and *in vivo* studies have demonstrated that DNA methylation is altered by exposure to toxic metals, including lead, arsenic and cadmium^47–49^. A recent study has demonstrated that early childhood lead exposure results in sex-dependent and gene-specific DNA methylation differences^50^. The expression of NLGN1 has been reported to be regulated by the methylation status of its gene promoter region^51^. Therefore, the Pb-induced inhibition of NLGN1 expression likely resulted secondary to Pb-induced an altered DNA methylation. A limitation of our study is the constraint in performing studies to address whether up-regulation of NLGN1 *in vivo* may have analogous rescuing effects.

In conclusion, low-level human equivalent GLE decreased NLGN1 protein expression, thus affecting the density and morphology of dendritic spines in hippocampus, likely inhibiting LTP and hence impairing learning and memory. Future studies could be profitably focused on the potential of NLGN1 to offer a therapeutic target to mitigate the Pb-induced effects on hippocampal synaptogenesis, and learning and memory deficits.

## Materials and Methods

### Animals

Animal procedures and experiments were carried out in strict accordance with the international standards of care guidelines, and were approved by the Institutional Animal Care and Use Committee of the Fourth Military Medical University. Sprague-Dawley rats were maintained on a 12-h/12-h light/dark cycle and in a temperature-controlled room at 24 °C with food and water available ad libitum.

### GLE model

Two weeks after arrival, female rats were individually housed and randomly divided into four groups: one control group and three GLE groups^52^. The control group received water, and the GLE groups received 0.005% (27 ppm Pb), 0.01% (55 ppm Pb) and 0.02% Pb acetate (109 ppm Pb) drinking solutions (Fisher Scientific, Pittsburgh, PA), respectively. Pb-adulterated drinking solutions were provided to the dams 2 weeks prior to mating to ensure blood Pb concentration stabilization and a constant Pb body burden throughout gestation, and up to PND10. Blood Pb concentrations were analyzed with electrothermal atomization atomic absorptionspectroscopy (AAS) on PNDs 0, 3, 7, 10, 14, 21, and 30. We performed morphologic and neurochemical experiments on PND 30. A previous study has shown that there were distinct differences between male and female infants and children in gestational Pb exposure model^53^; therefore, only male rats were used.

### Neuronal Culture and Transfection

Primary neurons were prepared from embryonic day18~19(E18~19) rat hippocampus as previously described by Craven *et al*.^54^. Hippocampi were collected by dissection on ice and dissociated with scissors. 0.5 ml of 2.5% trypsin was added and incubate for 20 min in a water bath at 37 °C, Gently remove trypsin solution and triturated the hippocampi. Plate the desired number of cells (1.5 * 10^6^ cells/ml) to dishes precoated with poly-L-lysine (0.5 mg/ml) (Sigma-Aldrich, USA) in serum-free neurobasal media supplemented with B27 and glutamax (GIBCOBRL, USA) then put it in incubator. At day *in vitro* (DIV) 3, add cytosine arabinoside (1-b-D-arabinofuranosylcytosine) to inhibit glial proliferation in a final concentration of 5 mM. Neurons were transfected with CMV-MCS-3FLAG-SV40-Neomycin (Genechem, Shanghai, China) at 4 mg per well for 6-well plates by Lipofectamine 2000 (Invitrogen) at DIV 6. Since during DIV 7–12 is the primary time period of dendritic spine growth^55^, for lead exposure, cultures were treated with lead acetate (1 μM, Sigma-Aldrich, USA) for 5 days from DIV 7 to DIV 12.

### Morris water maze tasks

The Morris water maze was divided into four quadrants of equal size and a cylindrical dark olive-green colored platform (10 cm in diameter) was placed in one of the quadrants (the target quadrant). Water temperature was held at 22 ± 1 °C. The swimming paths of the rats were tracked using a digital video camera suspended centrally above the pool and analyzed with the DigiBehave system (Jiliang Software Company, Shanghai, China). The mean value of the time that rats needed to find the platform was defined as the escape latency. The procedure consists of training for 5 days and testing for 1 day. On the training day, the platform was positioned in the middle of the southwest (SW) quadrant. The rats were released into the tank facing the tank wall at north (N), west (W), south (S) or east (E) quadrants in a predetermined pseudorandom order. A trial was terminated once the rat found the platform. And any rats that could not find the platform within 120 s were guided onto the platform and allowed to stay there for 30 s. On the testing day, the platform was removed from the pool; rats were released into the pool at NE position and allowed to swim freely for 2 min. The percentage of time that the animals spent in the SW quadrant in the probe trial was calculated.

### Lead concentration analysis

Blood samples (2 ml per animal) were collected in heparinized syringes and analyzed by a PerkinElmer 600 atomic absorption spectrometer (AAS)(PerkinElmer, USA). The hippocampus Pb concentrations were measured by a plasmaQuad3 plasma mass spectrograph (VG Elemental, UK) after tissue collection and digestion with an organic tissue solubilizer.

### Slice preparation and recording

Rats were decapitated between PND 28 and 35. The brains were quickly removed and immersed into cold oxygenated (95% O_2_/5% CO_2_) artificial cerebrospinal fluid (ACSF), The hippocampus was dissected free and transverse slices (400–500 μm) were cut with a sliding microtome. The slices were incubated for at least 1 h in ACSF at room temperature. Then one hippocampal slice was transferred to the recording chamber (BSC-HT Med. Sys., USA) continuously perfused at a rate of 1 ml/min with 30–32 °C ACSF saturated with 95% O_2_/5% CO_2_. Electrophysiological recording was conducted in only one slice per animal. After 1 h equilibration in the slice chamber, excitatory postsynaptic currents (EPSCs) were recorded. One bipolar stimulating electrode was located in the Schaffer/commissural fibers. The other recording electrode, a glass micropipette (resistance: 1–3 MΩ, tip diameter: 3–5 μm) filled with ACSF, was positioned in the dendrites of CA1 pyramidal cell. EPSCs were evoked by using 0.2–0.3 mA stimuli for 0.2 ms duration at 0.05 Hz. After recording the baseline responses for 10 min, LTP was induced by applying high-frequency stimulation (HFS) of 100 Hz for 1 s, testing with single shocks was repeated for at least 30 min after HFS. LTP was presented as the increase in EPSC in relation to the baseline response (100%) after tetanic stimulus application, and its amplitude was the mean of relative EPSC in 10–40 min.

### Golgi staining

Rats were anesthetized with an overdose of sodium pentobarbital (100 mg/kg, i.p.) and transcardially perfused with 0.9% saline. Next, the brains were placed directly into Golgi solution (10 g potassium chromate, 10 g mercuric chloride, 8 g potassium chloride and 1000 ml double-distilled water) where they remained in a foil-wrapped jar for 3 weeks. The solution was exchanged sequentially in 10, 20 and 30% sucrose solutions in light-protected jars to aid in maintaining histological structure. The brains were coronally sectioned at 100 μm thickness with a vibratome (VT1000S, Leica, Germany) and placed onto gelatin-coated glass slides. Slides were processed and covered with aluminum foil to prevent light contamination. Incubated slides in ammonium hydroxide for 10 min after rinsing. The slides were washed and incubated in Kodak Film solution for another 10 min and then rinsed for the last time. Next, the slides were dehydrated in ethanol, cleared in xylenes, and mounted using a resinous medium.

### Morphological analysis

Pyramidal neurons in the CA1 region of the hippocampus plate 58–63 were selected for analysis^56^. A Zeiss microscope with a motor stage equipped with transducers on the XYZ-axes was used to trace each neuron at low magnification (20X) for dendritic tree reconstruction and at high magnification (100X) for spine reconstruction at 0.5-μm or 0.1-μm increments, respectively. For each animal, three sections were used to generate nine z-stacks. An algorithm for tracing dendritic filaments (Imaris Bitplane) was used in autopath mode to reconstruct the entire dendritic tree starting from the soma of pyramidal neurons. Dendritic tracing originated from soma that having diameters ranging from 8 to 11 μm, and terminated once dendrite diameters became smaller than 0.6 μm. Sholl analysis was then performed on the three-dimensionally reconstructed neurons to calculate the number of intersections per each Sholl ring (20 μm interval) in order to gather information on the changes in dendritic tree complexity. Total length of basal dendrites was also measured for each neuron.

### Spine reconstruction and classification

Once the dendritic tree is reconstructed, the software (Imaris Bitplane) then reanalyzes dendritic segments for spines. Computer-generated seed points then label individual dendritic spines and following manual verification, quantification of spine density is performed. For spines to be included in our analyses, a maximum spine length and minimum spine end diameter were set at 2.5 μm and 0.4 μm, respectively. We only traced spines that were fully attached to dendritic segments and avoided spines whose structure was not completely visible. For spine density measurement, one terminal dendrite from the third order tip (minimum length 20 μm) of each selected neuron was used to count spines at a magnification of 100X. The results are expressed as number of spines/10 μm.

### Western blot analysis

Equal amounts of protein (50 μg) from the hippocampus were separated on 10% sodium dodecyl sulfate-polyacrylamide gels and blotted onto PVDF membranes (Invitrogen). After blocking by 5% non-fat dry milk, the membranes were incubated with antibodies for NLGN1 (Abcam, catalog ab186279, dilution ratio 1:200) and β-actin (Sigma, dilution ratio 1:1000) overnight at 4 °C. After washing, the membranes were incubated with horseradish peroxidase-conjugated secondary antibodies, and bands were visualized with an ECL system (Perkin Elmer). Band intensities were determined by software (ImageJ; National Institutes of Health, Bethesda, MD).

### Real-time fluorescence quantitative PCR

Hippocampal total RNAs was extracted with an RNA kit (Axygen, Silicon Valley, USA). The quality of the RNA (A260/A280) was 1.8–2.0 for all preparations. The primer OligodT was used to complete qPCR according to the manufacturer’s instructions (TransGene, Shanghai, China). The primer used is as follows: TTC AGT TTC TTG GGG TTC C-AAC CAC ACA GGA AGC ATA A for NLGN1. The 20 μL reaction pool of RTFQPCR was composed of: 10 μL of SYBR premix Extaq; 0.8 μL off orward and reverse primer each; 2 μL of cDNA template (10 time sdilution) and 6.4 μL of deionized water. For qPCR analysis, the levels of mRNA were quantified by FastStart Universal SYBR Green Master (Rox) (Roche, Basel, Switzerland) in an ABI 7500 (Applied Biosystems) Real-Time PCR System. All amplification reactions were conducted in triplicate. Relative expression ratios were calculated by the ∆∆Ct method, where Ct is the threshold cycle value^57^, the glyceraldehyde phosphatede hydrogenase housekeeping gene was used for normalization. The reaction procedure was set as one cycle of 95 °C for 10 s, 40 cycles of 95 °C 10 s, 60 °C 30 s, followed by the melting stage of 95 °C 10 s, 65 °C 60 s and 97 °C 1 s, then the cooling stage of 37 °C 30 s. Transcription levels were calculated as the amounts relative to that of GAPDH under the same conditions.

### Immunofluorescence and spine analyses of cultured neurons

Primary cultured hippocampus neurons were washed three times with PBS (pH 7.4) and fixed in cold absolute methanol for 20 min at 4 °C, and then permeabilized with 0.05% Triton X-100 for 30 min in ice bath and blocked with 5% bovine serum albumin (BSA) for 30 min at room temperature. Next, neurons were incubated with polyclonal anti-MAP2 (Abcam, catalog ab11267, dilution ratio 1:200) at 4 °C for overnight, followed by incubation with Alexa 488 (green)-conjugated anti-rabbit IgG (Invitrogen, dilution ratio 1:800) at room temperature for 2 h. Next, F-actin was labeled with TRITC-conjugated phalloidin (Sigma-Aldrich, catalog P1951, dilution ratio 1:500) at room temperature for 2 h, and rinsed again with PBS. Neurons were examined and photographed with an Olympus BX51 fluorescent microscope equipped with DP-BSW software (Olympus, Japan). In all studies, blanks were processed as negative controls, except that the primary antibodies were replaced with PBS. Spine analyses of cultured neurons were performed with at least 3 independent neuronal preparations on 2–4 independent coverslips each. All experiments included the full set of controls, and neurons were selected at random.

### Statistics

Each experiment was performed at least for three times. Data are presented as mean ± SD for at least three independent biological samples. The results were analyzed by one-way ANOVA followed by a Dunnett’s posttest for multiple comparisons. The variations in NLGN1 protein levels or spine densities caused by Pb or NLGN1 overexpression were determined with two-way ANOVA, followed by Bonferroni’s post-hoc analysis. All analyses were performed with the Statistical Package for the Social Sciences (SPSS 16.0) software. Levels of significance are indicated by the number of symbols, e.g., *p = 0.01 to <0.05; **p < 0.01. p < 0.05 was considered as the statistical difference.
